# Research on Predicting the Safety Factor of Plain Shotcrete Support in Laneways Based on BO-CatBoost Model

**DOI:** 10.3390/biomimetics9070394

**Published:** 2024-06-28

**Authors:** Haiping Yuan, Shuaijie Ji, Chuanqi Zhu, Lei Wang

**Affiliations:** 1 State Key Laboratory of Mining Induced Response and Disaster Prevention and Control in Deep Coal Mines, Anhui University of Science and Technology, Huainan 232001, China; zhuchuanqi2013@126.com (C.Z.); leiwang723@126.com (L.W.); 2 College of Civil Engineering, Hefei University of Technology, Hefei 230009, China; 15056020938@163.com

**Keywords:** laneway support, safety factor, CRITIC, CatBoost, Bayesian optimization

## Abstract

In general, the design of a safe and rational laneway support scheme signifies a crucial prerequisite for ensuring the security and efficiency of mining exploitation in mines. Nevertheless, the conventional empirical support system for mining laneways faces challenges in assessing the rationality of support methods, which can compromise the safety and reliability of the laneways. To address this issue, the safety factor was incorporated into research on laneway support, and a safety evaluation method for laneway support in line with the safety factor was established. In light of the data from a specific iron mine laneway in central China, the CRITIC method was employed to preprocess the sample data. Going one step further, a Bayesian algorithm was utilized to optimize the hyperparameters of the CatBoost model, followed by proposing a prediction model based on the BO-CatBoost model for evaluating laneway safety factors of plain shotcrete support. Furthermore, the performance indexes, such as the root mean square error (*RMSE*), the mean absolute error (*MAE*), the correlation coefficient (*R*^2^), the variance accounts for (*VAF*), and the a-20 index, were determined to examine the predictive performance of each proposed model. In contrast to the other models, the BO-CatBoost model demonstrated the optimal predictive output item for safety factors with the lowest *RMSE* and *MAE*, the largest *R*^2^ and *VAF*, and an appropriate a-20 index value of 0.5688, 0.4074, 0.9553, 95.25%, and 0.9167 in the test set, respectively. Therefore, the BO-CatBoost model was proven to be the most appropriate machine learning method that can more accurately predict the safety factor, which will provide a novel approach for optimizing laneway support design and laneway safety evaluation.

## 1. Introduction

A laneway is a passageway or strike excavated during underground mining, which is usually supported by rock or concrete to ensure safety and stability. With the depletion of shallow mineral resources, metal mines are gradually shifting towards deep mining. As a result, deep rock masses are subjected to a complex stress environment known as “three highs and one disturbance” [1,2]. The design of deep-rock laneway support has become a key issue in deep mining engineering. Nevertheless, the conventional empirical support system for mining laneways faces challenges in judging the rationality of support methods due to variations in ground pressure characteristics and rock mass grades across different areas within mines or even within the same mine [3]. This often leads to two extremes: excessive support strength increases costs and hampers excavation speed, while insufficient support strength fails to control surrounding rock deformation, resulting in a vicious cycle of “failure–recovery–failure” in the laneway [4]. Therefore, there is an urgent need to study new methods for evaluating laneway support that ensure safety and efficiency.

Currently, optimization methods for laneway support primarily rely on traditional engineering rock mass quality grading results to propose corresponding support schemes. Tang et al. [5] used RQD, RMR, and Q system grading methods to evaluate the rock mass quality of the laneway and optimized different support schemes and parameters through numerical modeling. Palmstrom et al. [6] questioned the parameters that made up the Q-classification system and explained its limitations when dealing with complex rock masses.

Although these traditional methods of engineering rock mass quality grading are simple and widely used in laneway support optimization research, they fail to fully consider geological characteristics such as high ground stress and ground pressure encountered in deep laneways. Additionally, certain factors are based on empirical judgment rather than objective criteria, greatly affecting evaluation reliability.

Given the limitations of traditional methods, numerous scholars have resorted to numerical simulation to compare and analyze various support schemes in order to identify the optimal combination of support parameters, which can reduce engineering costs and improve engineering quality. The optimization method based on numerical simulation has become a crucial research direction in geotechnical engineering. Wu et al. [7] made use of extension theory to evaluate the quality grade of rock mass, and on this basis, the rationality of the classification support design was verified through FLAC^3D^ numerical simulation. Chen et al. [8] classified rock mass quality based on the Hoek–Brown strength criterion and GSI method and established an empirical formula for the minimum safety factor of anchor rods through FLAC^3D^ numerical calculation. Zhang et al. [9] employed FLAC^3D^ numerical simulation to study the effects of anchor parameters and support modes on the deformation and plastic yield zone of surrounding rock laneways, thereby optimizing anchor support parameters such as diameter, length, spacing, etc. Zhu et al. [10] applied numerical simulation to study the mechanical behavior of surrounding rock under different anchor rod numbers and anchoring parameters and compared it with experimental results.

Numerical simulation methods have been extensively utilized in the field of laneway support; however, they do possess certain limitations. First, the simulation results are influenced by model assumptions and parameter settings, and it is necessary to conduct reasonable validation and calibration of the model. Additionally, deep-rock laneway support involves intricate problems in rock mechanics and structural mechanics, and its calculation process often involves a multitude of nonlinearities and multi-physical field couplings [11], which makes the calculation process of numerical simulation exceptionally complex. In certain special cases, the accuracy and reliability of the model still need to be improved.

With the rapid advancement of artificial intelligence, “digitization + artificial intelligence” has garnered significant attention from both domestic and international researchers. Machine learning algorithms have been extensively employed in diverse fields, including geotechnical engineering [12]. Gong et al. [13] simplified sample indicators through principal component analysis (PCA). On this basis, the particle swarm optimization algorithm was used to optimize the RBF neural network, and a tunnel-surrounding rock safety prediction model based on PCA-IRBF was established. Li et al. [14] developed a rock classification method suitable for high-stress tunnels based on BP neural networks; however, it suffered from slow convergence speed and susceptibility to local optima. The whale algorithm was employed by Zhou et al. [15] to optimize the support vector machine (SVM) for making classification predictions on 114 tunnel extrusion cases, resulting in improved prediction performance of the optimized SVM. 

Although individual machine learning methods as well as other combination algorithms possess unique advantages in solving nonlinear prediction problems, ensemble learning algorithms demonstrate higher accuracy and generalization ability compared to them [16,17]. Zhou et al. [18] utilized ten different machine learning algorithms such as support vector machines and random forests to predict rock burst levels. A comparative analysis of classification rate and Cohen’s Kappa accuracy revealed that ensemble learning models (RF, GBM) exhibited superior predictive performance. Niaz Muhammad Shahani et al. [19] employed extreme gradient-boosting machine learning algorithms to predict the uniaxial strength of rocks, and the XGBoost model showed better prediction accuracy. Huang et al. [20] used support vector machines, random forests, and CatBoost algorithms to predict reference evapotranspiration. Through comparative studies of model performance, the CatBoost model showed significant advantages in accuracy and generalization ability. 

Nevertheless, there are some shortcomings for the single-ensemble algorithm when making predictions. In the first place, it may not be able to find the optimal hyperparameter combination in terms of the single-ensemble algorithm due to the lack of optimization for hyperparameters, resulting in suboptimal model performances. In real-world applications, it is vital to optimize the hyperparameters, which can significantly improve the prediction accuracy and generalization ability of the model. In addition, many scholars choose to normalize the original data before model training to eliminate the influences of primary data dimensionality. However, the impact of an indicator’s importance on the convergence and predictive performance of the model tends to be ignored by simple normalized processing. The significance of different indexes may vary, but merely performing the straightforward normalization approach without considering the former is likely to lead to insufficient learning ability of the model for crucial indicators, affecting the predictive performance of the model.

In brief, there are the following predominant shortcomings and gaps within the research in the field of mine laneway support: Primarily, the traditional mine roadway support design is usually based on the empirical support systems, which have specific limitations and will tremendously affect the reliability of evaluation results. Subsequently, on account of the lack of a unified standard or consensus on displacement warning values for the excavation and support of mine laneways, there are evident research gaps in quantitatively evaluating the safety of mine laneway support. Moreover, the machine learning algorithms are broadly applied in the field of geotechnical engineering, especially in predicting geological disasters, but there are apparent research gaps in the optimization design and safety evaluation of mine laneway support.

In accordance with the aforementioned problem of insufficient research findings, the concept of the safety factor is introduced into the quantitative research and analysis of laneway plain shotcrete support in the current study, aiming to reasonably determine the laneway support parameters, optimize the support design, and quantitatively evaluate laneway safety. Furthermore, an innovative learning algorithm (BO-CatBoost model) is proposed for predicting the safety factors of laneway plain shotcrete support. First of all, the CRITIC method is adopted by the model to preprocess the sample data, which can not only eliminate the influences of dimensionality but also enable the sample data to more clearly reflect the importances of indicators. Subsequently, the CatBoost algorithm is integrated with the Bayesian optimization (BO) algorithm to optimize its hyperparameters. Ultimately, the five performance indexes, such as the root mean square error (*RMSE*), the mean absolute error (*MAE*), the correlation coefficient (*R*^2^), variance accounts for (*VAF*), and a-20 index, are determined to assess the predictive performance of each proposed model. In contrast to the other models, such as support vector regression (SVR), random forest (RF), and unoptimized categorical boosting (CatBoost), the BO-CatBoost model demonstrated the optimal predictive output item for safety factors with the lowest *RMSE* and *MAE*, the largest *R*^2^ and *VAF*, and appropriate a-20 index values of 0.5688, 0.4074, 0.9553, 95.25%, and 0.9167 in the test set, respectively. Consequently, the research findings have revealed that the BO-CatBoost model is equipped with superior predictive performance and generalization capability, which will provide an updated approach for optimizing mine laneway support and safety assessment.

## 2. Materials and Methods

### 2.1. Definition of Safety Factor

At present, there is a lack of standardized criteria or consensus regarding displacement warning values for laneway excavation, support, and production maintenance. This paper proposes an evaluation method aimed at ensuring the overall structural safety by analyzing the internal stress of the supporting structure. The safety factor of the laneway is defined as the ratio between internal stress intensity and ultimate allowable strength in order to quantitatively evaluate the safety and stability of laneways. The specific formula is as follows [21,22]:

(1) $$F_{s}=\frac{\sigma }{\left[\sigma \right]}$$

In Equation (1), $F_{s}$ is the safety factor, σ is the internal stress of the supporting structure, and $\left[\sigma \right]$ is the ultimate stress strength.

### 2.2. CRITIC Method

The CRITIC method is based on the comparative strength of evaluation indicators and the conflicts between indicators to comprehensively determine the objective weight of each indicator [23,24]. The specific steps to solve the objective weight by the CRITIC method are as follows:

(1) The evaluation matrix is constructed based on a total of m samples and n indicators, where $x_{ij}$ represents the value of the j-th evaluation indicator for the i-th sample.

(2) $$X={\left(x_{ij}\right)}_{m\times n}=\left[\begin{array}{cccc} x_{11} & x_{12} & \cdots & x_{1n}\\ x_{21} & x_{22} & \cdots & x_{2n}\\ \vdots & \vdots & \vdots & \vdots \\ x_{m1} & x_{m2} & \cdots & x_{mn} \end{array}\right]$$

(2) The indicators in X need to be standardized in order to mitigate the impact of dimension on their magnitude.

Favorable indicators:

(3) $$x_{ij}^{\prime }=\frac{x_{ij}-x_{min}}{x_{max}-x_{min}}$$

Inverse indicators:

(4) $$x_{ij}^{\prime }=\frac{x_{max}-x_{ij}}{x_{max}-x_{min}}$$

where $x_{min}$ is the minimum value for each indicator, $x_{max}$ is the maximum value for each indicator, and $x_{ij}$ is the indicator value.

(3) Calculating the coefficient of variation $\nu_{j}$

(5) $$\nu_{j}=\frac{\sqrt{\frac{1}{m-1}\;\sum_{i=1}^{m}{\left(x_{ij}^{\prime }-{\bar{x}}_{j}\right)}^{2}}}{\frac{1}{n}\sum_{i=1}^{n}x_{ij}^{\prime }}$$

(4) Calculating the coefficient $S_{j}$

(6) $$S_{j}=\sum_{i=1}^{n}\left(1-r_{ij}\right)$$

where $r_{ij}$ is the correlation coefficient between evaluation indexes i and j.

(5) Calculating the information content of indicators $C_{j}$

(7) $$C_{j}=\nu_{j}S_{j}$$

(6) Calculating the objective weight $\omega_{j}$

(8) $$\omega_{j}=\frac{C_{j}}{\sum_{i=1}^{n}C_{j}}$$

(7) Obtaining preprocessed sample data $x_{ij}^{f}$

(9) $$x_{ij}^{f}=\omega_{j}x_{ij}^{*}$$

### 2.3. CatBoost

**Figure 1:**
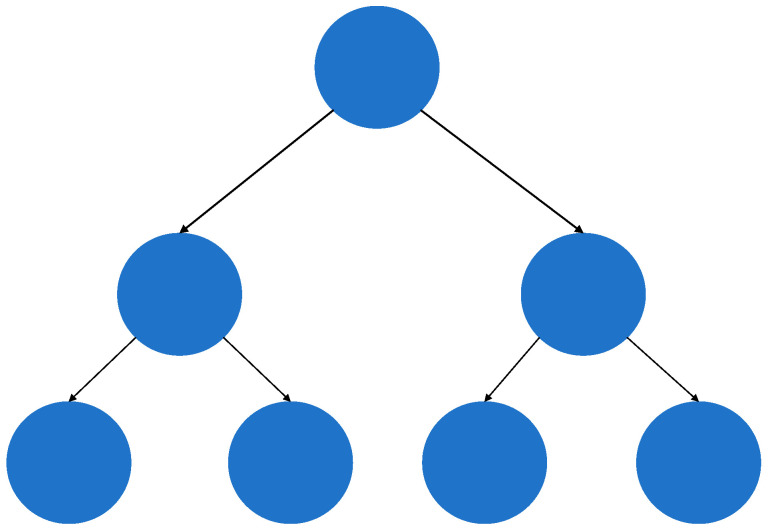
Schematic diagram of a symmetric full binary tree.

CatBoost is an improved GBDT (gradient-boosting decision tree) algorithm that can handle various types of data well, with strong robustness and excellent universality [25]. The conventional iterative process of GBDT is based on utilizing the same dataset to calculate the gradient of the current model and train to obtain the base learner based on this gradient. The aforementioned approach, however, may introduce estimation biases in the point-by-point gradients that result in the overfitting of the ultimately learned model. The CatBoost algorithm utilizes a symmetric tree as its base learner (refer to Figure 1), where the left and right subtrees are completely symmetrical and balanced [26]. This can ensure consistent split features in each layer during the iteration process, thereby reducing model complexity, improving prediction speed, and mitigating overfitting.

The GBDT algorithm uses the average value of labels as a criterion for node splitting; this method is called TBS. However, this approach causes information to be missing to a certain extent, leading to gradient bias and conditional migration problems. By incorporating a prior distribution term, CatBoost mitigates the impact of noise and low-frequency data on the data distribution, thereby enhancing the generalization ability of the model. The conversion formula is presented below.

(10) $$x_{i,k}=\frac{\sum_{j=1}^{p-1}\left[x_{\sigma j,k}=x_{pj,k}\right]Y_{\sigma j}+ap}{\sum_{j=1}^{p-1}\left[x_{\sigma j,k}=x_{pj,k}\right]Y_{\sigma j}+a}$$

In Equation (10), $x_{i,k}$ is the i-th sample feature of the k-th sample; $x_{\sigma j,k}$ is the i-th category feature of the j-th sample before the k-th sample; $Y_{\sigma j}$ is the label value of the j-th sample; p is the added prior term; and a is a weight coefficient greater than 0.

### 2.4. Bayesian Optimization Algorithm

In order to further improve the accuracy of the CatBoost algorithm in safety factor prediction, this study adopts a Bayesian algorithm to optimize the parameters of the CatBoost algorithm during the training process. It aims to identify the optimal parameter combination that best fits the algorithm, thereby maximizing prediction accuracy. The probabilistic surrogate model and the collection function are two important components of Bayesian optimization algorithms.

The probabilistic surrogate model estimates the distribution of the objective function by iteratively updating priors through a finite number of observation points [27]. This paper employs a Gaussian process as the probability proxy model, with its expression provided below:

(11) $$f\left(x\right)\sim GP\left(m\left(x\right),k\left(x,,x^{\prime }\right)\right)$$

where the mean function is $m\left(x\right)=E\left[f\left(x\right)\right]$ and the covariance function is $k\left(x,x^{\prime }\right)=E\left[\left(f\left(x\right)-m\left(x\right)\right)\left(f\left(x^{\prime }\right)-m\left(x^{\prime }\right)\right)\right]$.

The collection function is utilized for determining the next observation point and identifying the hyperparameter that maximizes model performance [28]. In this paper, the expected improvement (EI) function is employed as the collection function, while defining the improvement function I as follows:

(12) $$I\left(x\right)=max\left\{0,f_{t+1}\left(x\right)-f\left(x^{+}\right)\right\}$$

The probability density function of the random variable *I* is as follows:

(13) $$f\left(I\right)=\frac{1}{\sqrt{2\pi }\sigma \left(x\right)}\exp\left(-\frac{{\left(u\left(x\right)-f\left(x^{+}\right)-I\right)}^{2}}{2\sigma^{2}\left(x\right)}\right),I\geq 0$$

The expectation for the improvement value was obtained by integrating Equation (13):

(14) $$\begin{array}{c} E\left(I\right)=\int_{I=0}^{I=\infty }I\frac{1}{\sqrt{2\pi }\sigma \left(x\right)}\exp\left(-\frac{{\left(u\left(x\right)-f\left(x^{+}\right)-I\right)}^{2}}{2\sigma^{2}\left(x\right)}\right)dI\\ =\sigma \left(x\right)\left[\frac{u\left(x\right)-f\left(x^{+}\right)}{\sigma \left(x\right)}\Phi \left(\frac{u\left(x\right)-f\left(x^{+}\right)}{\sigma \left(x\right)}\right)+\varphi \left(\frac{u\left(x\right)-f\left(x^{+}\right)}{\sigma \left(x\right)}\right)\right] \end{array}$$

In Equation (14), $\Phi \left(\cdot \right)$ represents the cumulative distribution function of the standard normal distribution, and $\varphi \left(\cdot \right)$ represents the probability density function of the standard normal distribution. Equation (14) is further simplified:

(15) $$EI\left(x\right)=\left\{\begin{array}{c} \left(u\left(x\right)-f\left(x^{+}\right)\right)\Phi \left(Z\right)+\sigma \left(x\right)\varphi \left(Z\right),\;\sigma \left(x\right)>0\;\\ \;0,\;\sigma \left(x\right)=0\; \end{array}\right.$$

where $Z=\frac{u\left(x\right)-f\left(x^{+}\right)}{\sigma \left(x\right)}$.

## 3. Establishment of BO-CatBoost Model

### 3.1. CRITIC Method for Data Preprocessing

Due to the different dimensions of the original data, the importance of indicators cannot be accurately reflected by indicator data, which can potentially impact the predictive performance of the model [29]. Therefore, preprocessing of the original sample data is essential before making predictions with the model. Due to the different contributions of safety factor evaluation indicators to the final judgment, it is necessary to assign weights to the indicators in order to determine their contributions. This paper comprehensively considers the information content and correlation of safety factor prediction indicators and selects the CRITIC method to preprocess the evaluation indicators. The steps are as follows.

(1) The n evaluation indicators of m safety factor samples form the initial indicator data matrix $X={\left(x_{ij}\right)}_{m\times n}$.
(2) The data are standardized according to Formulas (3) and (4) $X^{*}={\left(x_{ij}^{*}\right)}_{m\times n}$.
(3) The comprehensive information contained in each indicator is calculated using Formulas (5)–(7).
(4) According to the comprehensive information, the comprehensive weight of each evaluation indicator is calculated using Formula (8).
(5) The processed data $X_{f}^{*}$ are obtained according to Formula (9).

### 3.2. BO-CatBoost Model

**Figure 2:**
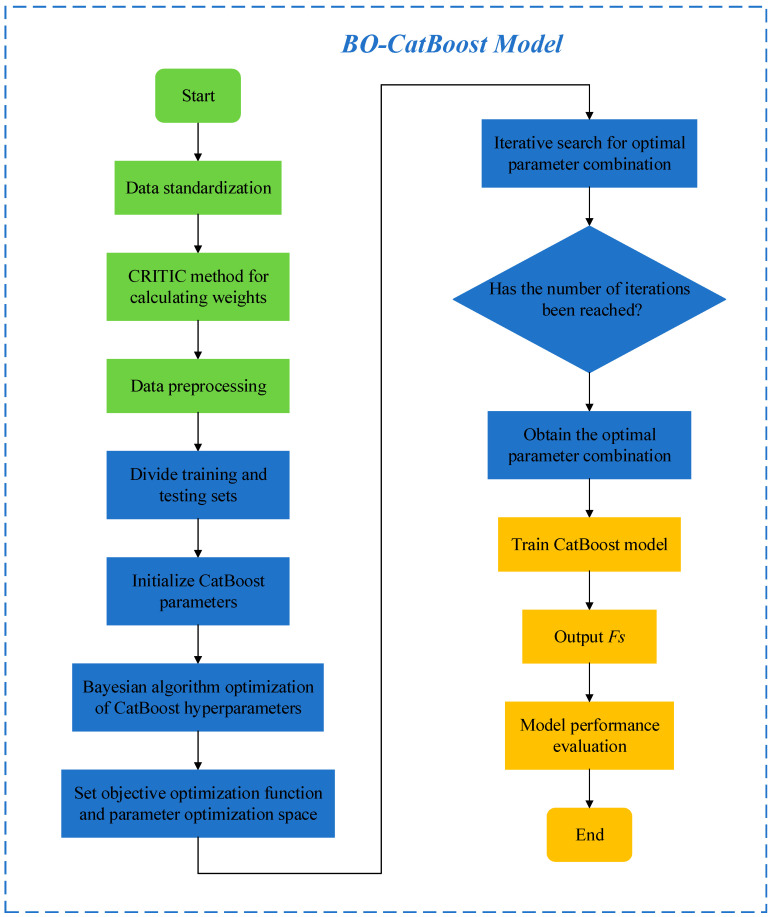
BO-CatBoost model flowchart.

After performing data preprocessing, the BO-CatBoost safety factor prediction model is established, and its process is illustrated in Figure 2. The specific steps are outlined as follows:

(1) Training model

The preprocessed data are utilized for training the CatBoost model, and the train set and the test set are randomly selected in definite proportions.

(2) Selecting parameter space

The hyperparameters that need to be optimized in the CatBoost algorithm are determined, and the search scope for each hyperparameter is defined.

(3) Setting the objective optimization function for Bayesian algorithm

The function takes the hyperparameters as the input item and returns the cross-validated performance indexes. The objective function in this paper represents the minimum mean squared error in order to find the most efficient combination of hyperparameters.

(4) Initializing the Bayesian optimizer

A Bayesian optimizer is initialized, and the objective function of the optimization, the hyperparameter space, and the optimized number of iterations are specified.

(5) Running the Bayesian optimization algorithm

The Bayesian optimization algorithm is run, and the optimal parameter combination is found by means of optimizing the hyperparameters continuously. After each iteration, the model of the Bayesian optimizer is updated based on the returned values of the objective function. Afterwards, it is determined whether the maximum number of iterations is reached, and the parameter combination that allows the CatBoost algorithm to achieve the optimal performance is output.

(6) Training the optimal model

The optimal combination of parameters is input into the CatBoost algorithm model, and the final prediction model of the *Fs* is obtained through training.

(7) Model evaluation

The performance of the final trained model is evaluated on the test set, and the regression evaluation indexes are applied to evaluate the predictive performance of the model.

## 4. Application: Case Studies

This paper focused on a large iron ore mine located in central China as the research context. With the increasing depth of mining, the geological conditions within this mine became more complex, leading to the emergence of ground pressure characteristics. During the mining process, rock caving and other hazardous conditions were prone to occur, which seriously threatened the safety of the operation.

### 4.1. Establishment of Safety Factor Evaluation Indicators

**Figure 3:**
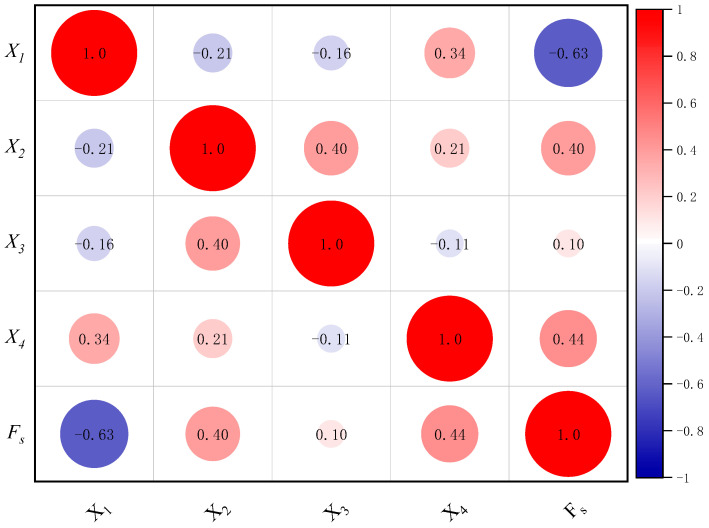
Correlation matrix heatmap.

**Figure 4:**
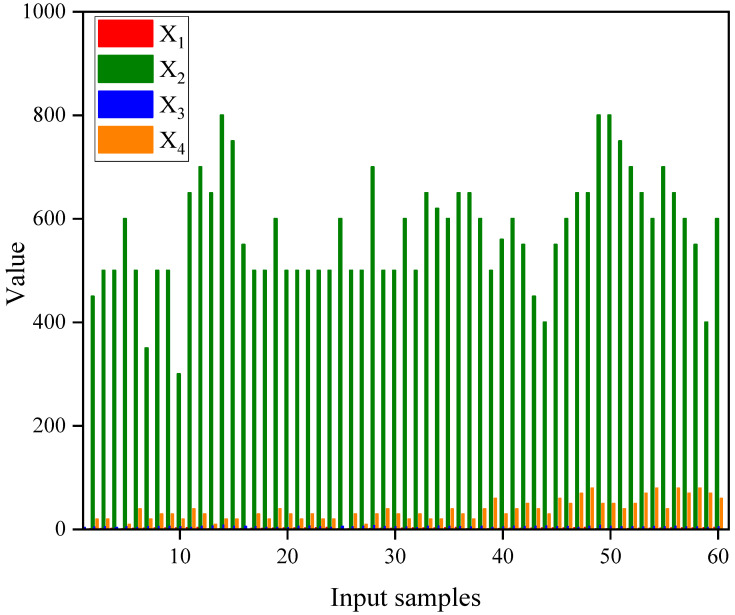
Histogram of input data.

The support of laneways, as the fundamental component of the entire mining system, was influenced by a multitude of factors. This paper referred to relevant research findings [30,31] and comprehensively selected four key factors that impacted the safety factor of laneway plain shotcrete support as evaluation indicators for laneway safety factor: refinement of rock mass rating ($X_{1}$), burial depth ($X_{2}$), laneway span ($X_{3}$), and support thickness ($X_{4}$). In light of the data from a specific iron mine laneway in central China, the BO-CatBoost model was employed to predict the safety factor. To be specific, a random seed was utilized to disrupt the sample dataset, with 48 groups of data as the training set and the remaining 12 groups as the test set. The original data are presented in Table 1, and the processed data are shown in Table 2. Additionally, $X_{1}$, $X_{2}$, $X_{3}$, and $X_{4}$ are used as inputs to the model, and $F_{s}$ is used as the output of the model. The correlation matrix heatmap is shown in Figure 3, and the histogram of the input data is shown in Figure 4.

### 4.2. Optimization Results of Bayesian Algorithm

The Bayesian optimization algorithm was employed in this study to optimize the parameters of CatBoost, with the selection of root mean square error as the objective function for optimization. The parameter optimization interval and results are presented in Table 3.

### 4.3. Comparison Algorithm Selection

The support vector regression (SVR), random forest (RF), and unoptimized CatBoost models, commonly employed in machine learning algorithms, were chosen as comparative algorithms to construct the prediction model for the safety factor of laneway plain shotcrete support in order to validate the effectiveness and reliability of the proposed model.

#### 4.3.1. SVR

Support vector regression (SVR) is a regression algorithm that is based on the principles of support vector machines, whose basic concept is to map the input sample data to a high-dimensional space through a mapping function $\Phi \left(x\right)$ to solve nonlinear problems [32]. Subsequently, a linear model is constructed to estimate the regression function in this high-dimensional space.

Assuming that the training sample $\left\{\left(x_{i},y_{i}\right)|x_{i}\in R,y_{i}\in R,i=1,2,\ldots ,n\right\}$, $x_{i}\in R$ is the input of the training sample and $y_{i}\in R$ is the output of the training samples, the SVR objective function is expressed as follows:

(16) $$min_{w,b}\left\{\frac{1}{2}{||w||}^{2}+C\overset{n}{\underset{i=1}{\sum }}\left(\xi_{i}+\xi_{i}^{*}\right)\right\}$$

The constraints include the following:

(17) $$\left\{\begin{array}{c} y_{i}-w\Phi \left(x_{i}\right)-b\leq \varepsilon +\xi_{i}^{*}\\ w\Phi \left(x_{i}\right)+b-y_{i}\leq \varepsilon +\xi_{i}^{*}\\ \xi_{i},\xi_{i}^{*}\geq 0,i=1,2,\ldots ,n \end{array}\right.$$

where C is the penalty factor; $\xi_{i}$ and $\xi_{i}^{*}$ are the relaxation variables; w and b are the normal vector and intercept of the hyperplane, respectively; and ε is the allowable error.

By introducing the Lagrange multipliers α and $\alpha^{*}$, the SVR optimization problem can be transformed into a dual problem, and the SVR prediction function can be obtained by solving the dual problem:

(18) $$f\left(x\right)=\overset{n}{\underset{i=1}{\sum }}\left(\alpha^{*}-\alpha \right)\kappa \left(x,x_{i}\right)+b$$

where $\kappa \left(x,x_{i}\right)$ is the kernel function. In this paper, the radial basis function is used as the kernel function.

#### 4.3.2. RF

Random forest (RF) is an ensemble algorithm proposed by Breiman in 2001 [33]. RF adopts a bootstrap resampling method to randomly select sample subsets from sample data as training samples. It constructs decision trees based on these data and repeatedly samples to establish a large number of decision trees, ultimately forming a random forest. Finally, through voting or averaging, the prediction results of each decision tree are combined to obtain the final predictions. This approach can reduce the dependence of the model on all feature values in the training samples and improve the generalization performance of the model.

### 4.4. Selection of Model Performance Evaluation Indicators

This study selected five common evaluation indicators in regression problems as model performance evaluation indicators, namely the root mean square error (*RMSE*), mean absolute error (*MAE*), correlation coefficient (*R*^2^), variance accounts for (*VAF*), and a-20 index. The *RMSE* reflected the sample standard deviation of the discrepancy between predicted and true values, while the *MAE* represented the average absolute error between predicted and true values. In nonlinear fitting, the smaller values of *RMSE* and *MAE* indicated higher degrees of fit and greater prediction accuracy. The *R*^2^ was utilized to measure how well the regression model aligns with observed data, and the closer *R*^2^ was to 1, the better the model fit the information. The *VAF* index represents the percentage between the variance of true and predicted values and the variance of predicted values [34]. The closer the *VAF* value is to 100%, the better the predictive ability of the model is. The *a-20 index* represents the ratio of the number of samples with a predicted deviation of ±20 from the true value to the number of samples [35]. The calculation formulas of these evaluation indicators are as follows:

(19) $$RMES=\sqrt{\frac{1}{n}\sum_{i=1}^{n}{\left(y_{i}-{\hat{y}}_{i}\right)}^{2}}$$

(20) $$MAE=\frac{1}{n}\sum_{i=1}^{n}\left|\left(y_{i}-{\hat{y}}_{i}\right)\right|$$

(21) $$R^{2}=1-\frac{\sum_{i=1}^{n}{\left(y_{i}-{\hat{y}}_{i}\right)}^{2}}{\sum_{i=1}^{n}{\left(y_{i}-{\bar{y}}_{i}\right)}^{2}}$$

(22) $$VAF=\left[1-\frac{var\left(y_{i}-{\hat{y}}_{i}\right)}{var\left({\hat{y}}_{i}\right)}\right]\times 100\%$$

(23) $$a-20\;index=\frac{m20}{N}$$

where n is the number of samples, $y_{i}$ is the true value, ${\hat{y}}_{i}$ is the predicted value, ${\bar{y}}_{i}$ is the mean of the true values of the sample, m20 represents a dataset with a ratio of true to predicted values between 0.80 and 1.20, and N is the number of datasets.

### 4.5. Performance Analysis of Safety Factor Prediction Model 

**Figure 5:**
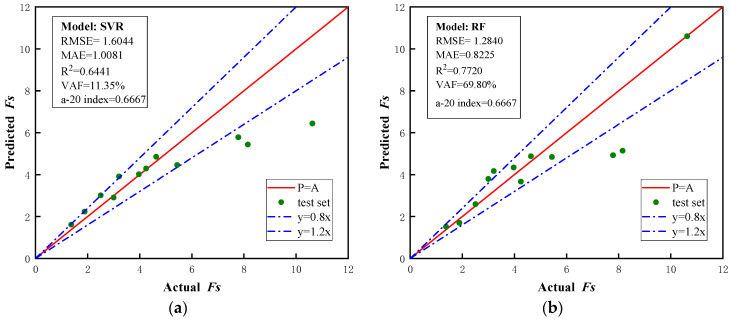

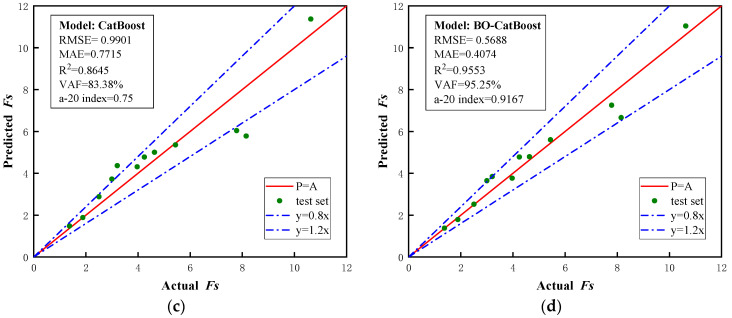
Prediction results of different model test sets: (**a**) SVR model prediction results; (**b**) RF model prediction results; (**c**) CatBoost model prediction results; (**d**) BO-CatBoost model prediction results.

As shown in Figure 5, the predicted and actual safety factor values from the SVR model, RF model, CatBoost model, and BO-CatBoost model are presented. It is noteworthy that the scatter plot was close to the diagonal P = A, indicating better prediction results. The performance indexes were employed to assess the learning ability and prediction capability of the model test set.

The prediction results of the SVR model are depicted in Figure 5a and exhibited significant deviation. The *RMSE*, *MAE*, *R*^2^, *VAF*, and a-20 index values for the test sets were 1.6044, 1.0081, 0.6441, 11.5%, and 0.6667, respectively, indicating substantial disparities between the predicted results of the SVR model and actual $F_{s}$ values. Consequently, it was concluded that the generalization ability of the SVR-based laneway safety factor prediction model was poor, and the model was unreliable.

The prediction results of the RF model are shown in Figure 5b. In comparison to the SVR model, the RF model demonstrated enhanced accuracy in predicting $F_{s}$ values. The *RMSE*, *MAE*, *R*^2^, *VAF*, and a-20 index values for the test sets were 1.2840, 0.8225, 0.7720, 69.80%, and 0.6667, respectively, indicating that the predicted values of the entire test set were closer to the true $F_{s}$ values. However, for individual sample points, the bias of the RF model was still relatively large.

The prediction results of the CatBoost model and BO-CatBoost model are presented in Figure 5c and Figure 5d, respectively. The CatBoost model demonstrated *RMSE*, *MAE*, *R*^2^, *VAF*, and a-20 index values of 0.9901, 0.7715, 0.8645, 83.38%, and 0.75, respectively, while the BO-CatBoost model exhibited respective values of 0.5688, 0.4074, 0.9553, 95.25%, and 0.9167, indicating its excellent predictive performance. However, upon optimizing the hyperparameters of the CatBoost model through the Bayesian algorithmic approach, both the *RMSE* and *MAE* values were significantly reduced from their initial values of 0.9901 and 0.7715 to 0.5688 and 0.4074, respectively. In the meantime, the values of *R*^2^, *VAF*, and the a-20 index of the CatBoost model improved from 0.8645, 83.38%, and 0.75 to 0.9553, 95.25%, and 0.9167, respectively. This indicated that the Bayesian algorithm optimization played a crucial role in enhancing the predictive performance of the CatBoost model.

**Figure 6:**
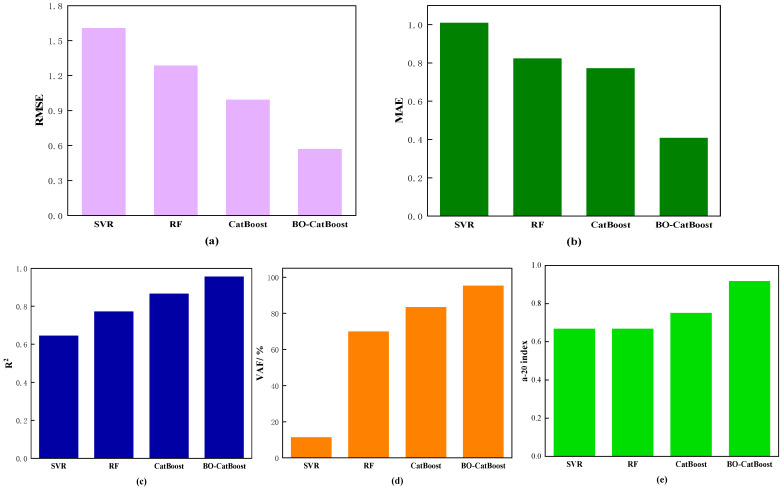
Model evaluation indicators: (**a**) *RMSE*; (**b**) *MAE*; (**c**) *R*^2^; (**d**) *VAF*; (**e**) a-20 index.

The performance index values of different models on the test set are summarized in Figure 6 in order to compare their performance more intuitively. The predictive ability of the four models on the test set was ranked from low to high as follows: SVR < RF < CatBoost < BO-CatBoost. The aforementioned evidence demonstrated that the CatBoost model combined with Bayesian optimization exhibited superior predictive performance and generalization capabilities, and the prediction results were more reliable.

**Figure 7:**
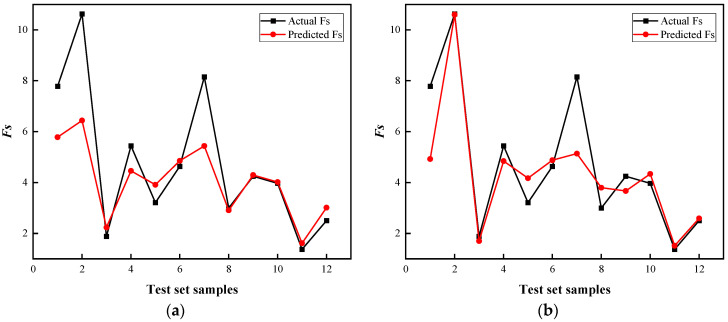

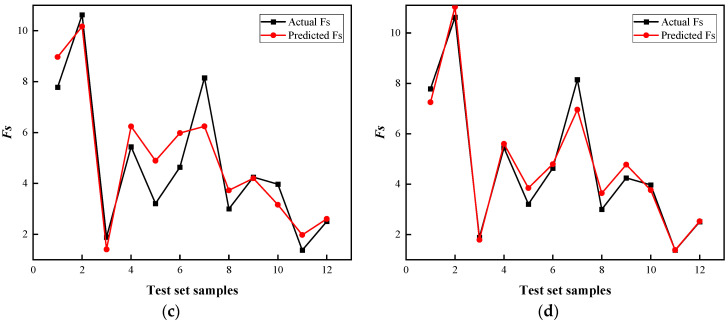
Prediction results of four models: (**a**) SVR model; (**b**) RF model; (**c**) CatBoost; (**d**) BO-CatBoost model.

**Figure 8:**
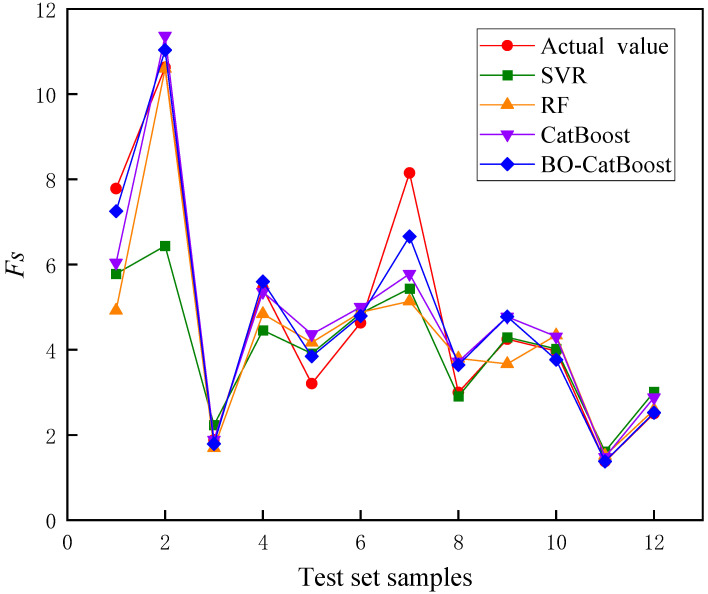
Comparison of the prediction results of different models.

The prediction results of the four models for the test set are shown in Figure 7, and the prediction results of different models are shown in Figure 8. Subsequently, Equation (24) is employed to compute the error between the predicted value and the actual value.

(24) $$Error=Fs_{p}-Fs_{A}$$

**Figure 9:**
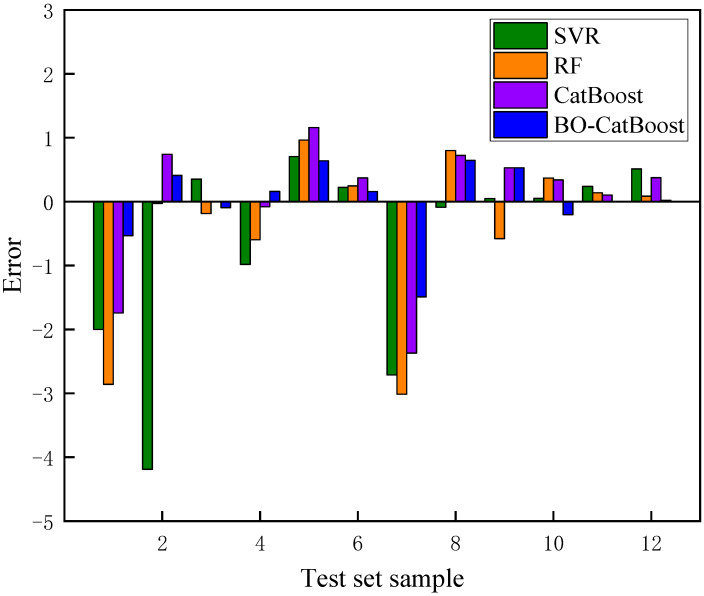
Prediction error results for four algorithm models on the test set.

In Equation (24), $Fs_{p}$ represents the predicted value of the safety factor, and $Fs_{A}$ is the actual value of the safety factor. Figure 9 shows the prediction errors corresponding to four different algorithm models. 

The analysis of Figure 7, Figure 8 and Figure 9 revealed that notable disparities existed in the accuracy levels of these four models. It is important to highlight that the SVR model exhibited the largest deviation from the actual $F_{s}$ values, with a maximum absolute error exceeding 4. This finding highlighted that the SVR model was not suitable for the prediction of laneway safety factor and may pose potential risks to engineering safety.

Compared with the SVR model, the RF model demonstrated an enhanced accuracy in predicting $F_{s}$ values; however, its maximum absolute error was still close to 3. On the other hand, the CatBoost model provided more precise predictions, with a maximum absolute error of 2.3. Meanwhile, the maximum absolute error of the BO-CatBoost model was further reduced to 1.4, while the maximum absolute errors of the other test samples were controlled within 1. Consequently, among the four models considered in this study, the BO-CatBoost model exhibited the best predictive performance.

In summary, the BO-CatBoost model exhibited superior generalization ability and reliable performance, making it well-suited for practical mine engineering applications. It was worth noting that among the prediction results of the BO-CatBoost model on the test set, only one test sample had an absolute error exceeding 1, with a predicted value lower than the actual value. This can be attributed to the fact that outliers in input variables may provide extreme information to the established predictive model. Therefore, it is necessary to incorporate actual field processing data from different mines when training the model. However, the overall results demonstrated that the proposed BO-CatBoost model could effectively and accurately predict the safety factor of a plain shotcrete support.

### 4.6. Feature Importance Analysis

**Figure 10:**
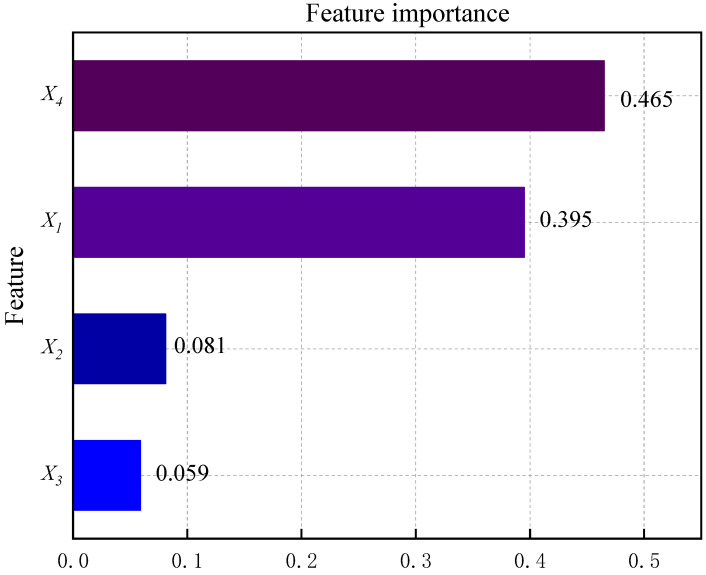
Indicator importance analysis from the BO-CatBoost model.

By utilizing the feature importance module within the CatBoost model, we calculated the importance level of each indicator. Figure 10 illustrates the importance of different evaluation indicators for predicting $F_{s}$ based on the BO-CatBoost model.

According to the results for the importance of features in Figure 10, the support thickness and the refinement of rock mass rating in the mining area were the most prominent indicators. Furthermore, the remaining features were arranged in descending order of importance, including the depth and span of the laneway. It is worth mentioning that laneway burial depth and span held lower importance, which confirmed that a good rock mass quality grade and reasonable support thickness in the mining area can weaken the adverse effects of laneway burial depth and span and ensure the basic stability of mining laneways. Consequently, in terms of the design and support work of mine laneway stability, the essential task is to focus on the evaluation of the quality rank of mining rock mass and to set different support thicknesses with respect to various laneway burial depths and spans, so as to achieve economic and effective mine laneway support design.

## 5. Conclusions

In this study, the safety factor was incorporated into the quantitative research and analysis of laneway support, with the adoption of the CRITIC method for preprocessing sample data to enhance the clarity in reflecting indicator importance. To be more specific, the Bayesian algorithm was utilized to optimize the hyperparameters of the CatBoost model, and the optimal prediction model for the safety factors of mining laneways, namely, the BO-CatBoost model, was established. Furthermore, the performance indexes, such as the root mean square error (*RMSE*), the mean absolute error (*MAE*), the correlation coefficient (*R*^2^), the variance accounts for (*VAF*), and the a-20 index, were determined to assess the predictive performance of each proposed model. The main conclusions can be presented as follows:

(1) In this paper, the safety factor was introduced into the quantitative research and analysis of roadway support, which has provided an updated research method for rationally determining the laneway support parameters, optimizing support design, and quantitatively evaluating the safety of laneways.
(2) In contrast to the rest of the models, such as the SVR, the RF, and the CatBoost models, the BO-CatBoost model demonstrated the optimal predictive output item for safety factors with the lowest *RMSE* and *MAE*, the largest *R*^2^ and *VAF*, and an appropriate a-20 index, with values of 0.5688, 0.4074, 0.9553, 95.25%, and 0.9167 in the test set, respectively. Thus, the BO-CatBoost model is found to be the best machine learning method that can most precisely predict the *Fs*.
(3) Compared with the unoptimized CatBoost model, the values of *RMSE* and *MAE* in the BO-CatBoost model decreased from 0.9901 and 0.7715 to 0.5688 and 0.4074, respectively. Moreover, the values of *R*^2^, *VAF*, and the a-20 index of the BO-CatBoost model improved from 0.8645, 83.38%, and 0.75 to 0.9553, 95.25%, and 0.9167, respectively. This indicates that the optimization of the Bayesian algorithm plays a crucial role in enhancing the predictive performance of the CatBoost model.
(4) According to the analysis of feature importance, the thickness of support and the refinement rank of rock mass quality in the mining area emerged as the two most critical elements for predicting the safety factors of mining laneways. Consequently, in terms of the design and support work of mine laneway stability, the essential task is to focus on the evaluation of the quality rank of mining rock mass and to set different support thicknesses with respect to various laneway burial depths and spans, so as to achieve economic and effective mine laneway support design.
(5) It is worth mentioning that expanding the dataset is beneficial for reducing the impact of extreme information, thereby improving the prediction accuracy of the model. Currently, there are insufficient practical cases of optimizing mine laneway support in existing mines, and the expansion of the dataset needs to be followed up with further research and discussion.

## Figures and Tables

**Table 1:** Original data of laneway shotcrete support.

Number	$X_{1}$	$X_{2}$/m	$X_{3}$/m	$X_{4}$/mm	$F_{s}$
1	1	500	4	0	4.91
2	1.5	450	4.5	20	4.868
3	1.8	500	5	20	3.631
4	2	500	4	0	0.825
⋯⋯					
59	3.5	400	4	70	3.988
60	4	600	5	60	2.997

**Table 2:** Processed data.

Number	$X_{1}$	$X_{2}$/m	$X_{3}$/m	$X_{4}$/mm	$F_{s}$
1	0.282	112.5	0.74	0	4.91
2	0.423	101.25	0.8325	6.16	4.86
3	0.5076	112.5	0.925	6.16	3.631
4	0.564	112.5	0.74	0	0.825
⋯⋯					
59	0.987	90	0.74	21.56	3.988
60	1.128	135	0.925	18.48	2.997

**Table 3:** CatBoost algorithm parameter search range and optimal value.

Parameters	Implication	Search Range	Optimal Value
iterations	Maximum number of iterations	[100, 1000]	681
learning_rate	Learning rate	[0.01, 0.1]	0.1
depth	The maximum depth of the tree	[4, 10]	4
l2_leaf_reg	L2 regularization to reduce overfitting	[1, 10]	1
random_strength	Disturbance term of feature splitting information gain to avoid overfitting	[1, 10]	10

## Data Availability

Data will be made available upon request.
